# Evaluation of the Efficacy of Different Pterygium Surgeries in Polish Caucasian Population

**DOI:** 10.1155/2022/1641350

**Published:** 2022-04-15

**Authors:** Marcin Palewski, Agnieszka Budnik, Łukasz Lisowski, Joanna Konopińska

**Affiliations:** Department of Ophthalmology, Medical University of Białystok, Białystok 15-089, Poland

## Abstract

The aim of the study was to compare the efficacy of the two most commonly used surgical methods for pterygium removal in the Polish population, conjunctival autograft versus amniotic membrane transplantation, and to evaluate the postoperative recurrence rate. A retrospective analysis of the medical records was conducted, including 65 patients who underwent surgery for primary or recurrent pterygium at an ophthalmology clinic in Bialystok, Poland, between 2016 and 2020. Surgical success (no regrowth) was achieved in almost half of the amniotic membrane patients (44%) and in most of the conjunctival autograft patients (79%), with statistical significance. The odds of successful surgery were 79% lower for subjects with amniotic membranes than for those with conjunctival autografts (OR with 95% CI = 0.21 (0.05; 0.94); *p*=0.045). Our study confirms that the conjunctival autograft surgeries present more favorable success rates in Polish Caucasian population when compared to procedures involving amniotic membranes.

## 1. Introduction

Pterygium is a common disorder of the anterior eye surface. According to population studies, the prevalence of pterygium ranges from 1% to 30% [1–3]. It is a fibrovascular degenerative lesion of the ocular conjunctiva that presents clinically as triangular-shaped growth consisting of conjunctival epithelium and hypertrophied subconjunctival connective tissue, with its apex facing the cornea. The ocular conjunctiva is the most common location of the lesion, located in the projection of the palpebral fissure from the nasal side. Major mechanisms of pterygium formation are triggered as a result of changes in the local homeostasis of the ocular surface, including the formation of proliferative limbal stromal cell (LSC) clusters, epithelial metaplasia, formation of active fibrovascular tissue, inflammation, and disruption of Bowman's layer along the infiltrating pterygium apex [4]. In severe cases, pterygium can grow into the central part of the cornea, potentially resulting in irregular corneal astigmatism, possibly resulting in decreased visual acuity, ranging from a significant decrease in visual acuity to loss of vision [5].

The most important risk factors for the development of pterygium and recurrences may include ethnicity, geographic latitude of residence and associated sun exposure [6], male sex, older age, smoking, rural origin, and darker skin complexion [3]. Other factors include chronic irritation from dust and air pollutants and anterior ocular surface surgery [7]. Viral aetiology, herpes simplex virus, human papilloma virus, cytomegalovirus, inflammation [8], and hereditary predispositions [9] are also among the risk factors for the development of pterygium [10]. Although the pathogenesis of pterygium is not well understood, it is known to be related to ultraviolet (UV) radiation. By inducing reactive oxygen species, UVA causes indirect DNA damage and activates transcription factors that regulate the expression of many genes involved in extracellular matrix changes [8, 11].

Pterygium can only be treated surgically. The main indications for surgical removal of a pterygium include deteriorating vision resulting from growth, increasing astigmatism, or recurrent inflammation. It is important to note that there is no “perfect” surgical method that guarantees no pterygium recurrence after removal. The most commonly used surgical techniques include conjunctival autografting, transposition of a pedicled conjunctival flap, and amniotic membrane transplantations [12–18]. Adjuvant therapy in the form of antimetabolites, antiangiogenic agents, and radiation is used to reduce the risk of recurrence [19].

There are studies in the literature evaluating the effectiveness of conjunctival autograft versus amniotic membrane suturing that have been evaluated in Turkish [20], Chinese [21, 22], Brazilian, or Indian [23] populations, but there are no such reports relating to Caucasian populations to date. There have been no such reports concerning the Polish population in the past. The aim of our study was to compare the efficacy of the two most commonly used surgical methods for pterygium in the Polish population, conjunctival autograft versus amniotic membrane suturing, and to evaluate the postoperative recurrence rate. According to our best knowledge, this is the first study of its kind in Central and Eastern European populations.

## 2. Materials and Methods

A retrospective analysis of the medical records was conducted for 65 patients who underwent surgery for primary or recurrent pterygium at an ophthalmology clinic in Bialystok, Poland, between 2016 and 2020. The study was approved by the Local Bioethics Committee of the Medical University of Bialystok and was conducted in accordance with the principles of the Declaration of Helsinki. The indication for the procedure was the presence of a nasally located pterygium of at least grade 1 [24].

Exclusion criteria included symblepharon, conjunctival scarring, and uncompensated glaucoma, which might require a filtering procedure in the future. Patients with a 6-month follow-up period were eligible for analysis.

Prior to the procedure and at each follow-up visit after the procedure, each patient underwent a complete ophthalmological examination, including anterior segment photography of the eye in a lamp (Led Digital Vision HR, SL9900 ZOOM-D, C. S. O. Italy), and measurement of best-corrected visual acuity (BCVA) and intraocular pressure (IOP) using the Goldmann applanation tonometer (GAT; Haag-Streit, Bern, Switzerland). To ensure a similar quality in all photographs, the diffuse lamp light was set to a width of 2 mm, and the height was adjusted to the edges of the dilated pupil at a 45° angle to the lens surface, at a 16x magnification. The photograph is focused on the cornea. The degree of pterygium progression was assessed as previously described [25].

Postoperative visits occurred 1 week, 2 weeks, 1 month, 3 months, 6 months, and 12 months after surgery. Corneal healing and the presence of possible recurrence were evaluated at the postoperative visits, in addition to the tests mentioned earlier. Pterygium recurrence was defined as growth greater than 1 mm beyond the corneal limbus 6 months after surgery.

Informed consent was obtained from all patients prior to the procedure. All procedures were performed by two experienced surgeons (A.B. and L.L.) under local anesthesia, according to a previously described technique [12, 23].

### 2.1. Excision with Conjunctival Autograft

After removal of the pterygium head and body, the scleral bed at the site of the removed pterygium was covered with a fragment of the patient's conjunctiva taken from the superior temporal quadrant of the same or the other eye, with special care to avoid interrupting the conjunctiva in the middle. Each time a conjunctival graft was collected, Tenon's tissue was carefully cleaned because it constitutes a source of fibroblast proliferation and a risk factor for pterygium regrowth. The graft was 1 mm larger on each side than on the dissected scleral bed. 8/0 absorbable sutures (Novosyn® 90/10; Braun) were used for conjunctival graft fixation.

### 2.2. Suturing the Amniotic Membrane

After removal of the head of the pterygium and cleaning of the sclera, a flap of amniotic membrane of size corresponding to the locus of the excised pterygium, increased by 1 mm, was sutured onto the resulting bed. Amniotic membrane was laid down with its epithelial side facing up. A flap of the drained amniotic membrane was cut to fit the scleral pocket shape and size. Excess transplanted tissue was cut off with scissors and then fixed with absorbable sutures (Novosyn® 90/10, Braun).

A dressing contact lens was applied to the cornea to improve graft adhesion and to reduce postoperative pain. All patients in both groups received moxifloxacin three times daily (Levomer, Adamed Pharma S.A) for the first week and dexamethasone three times daily (Dexafree, Bausch, and Lomb) from the time of corneal epithelialization at the site of the removed pterygium (2 days after the surgery on average), with gradual tapering for 3 months after the surgery.

## 3. Statistical Analysis

The relationships between group and qualitative variables were analyzed using Fisher's exact test. The number and percentage of observations and the odds ratio with a 95% confidence interval for the specific event occurrence were reported. The values of quantitative variables were compared between the groups using the Mann–Whitney *U* test. Differences between medians with 95% confidence intervals have also been reported. These calculations were performed using *R* statistical software version 4.1.2, with an assumed significance level of *α* = 0.05.

A post hoc analysis of the observed power was performed to assess the success of the surgery, resulting in 0.54 for this study, with *α* = 0.05. Post hoc analysis was performed using the *G* Power software, version 3.1.9.4.

## 4. Results

Women constituted a minority in both study groups (22% and 37%, respectively; *p*=0.479). The respondents in the two groups did not differ significantly by age (*p*=0.927) or follow-up time (*p*=0.127). Pterygium in the right eye was present in 22% of the subjects with amniotic membranes and in 33% of the subjects with conjunctival autografts (*p*=0.707). The chance of primary pterygium occurrence was lower by 90% in subjects with amniotic membrane than in those with conjunctival autograft (33% vs. 83%; OR with 95% CI = 0.10 (2.27; 53.27); *p*=0.005), and the chance of recurrent pterygium was 11 times higher in subjects with amniotic membrane than in those with conjunctival autograft (67% vs. 15%; OR with 95% CI = 11.00 (2.27; 53.27); *p*=0.003). Surgical success (no regrowth) occurred in almost half of the amniotic membrane patients (44%) and in most of the conjunctival autograft patients (79%), with a statistically significant relationship. The odds of successful surgery were 79% lower for subjects with amniotic membranes than for those with conjunctival autografts (OR with 95% CI = 0.21 (0.05; 0.94); *p*=0.045) (Table 1).

## 5. Discussion

In the literature, the incidence of pterygium varies considerably among countries [26]. The prevalence can reach 22% in equatorial areas and less than 2% at latitudes above 40° (0.9% in the German population) [7]. It is important to note that there is no “gold standard” in pterygium surgery. Postoperative recurrence is a significant problem in ophthalmic practice. In our study, we presented the effectiveness of pterygium treatment using two surgical methods: conjunctival autograft versus amniotic membrane suturing in the Polish population. Our results corroborate those of other studies, in which greater efficacy in preventing pterygium recurrence was observed with conjunctival autografts [22]. In our study, the recurrence rate for both surgical methods (21.2% and 55.6% for autograft and amniotic membrane suturing, respectively) was higher than that reported by other authors, which may be due to the absence of adjuvants.

Removal of the pterygium with conjunctival autograft involves covering the sclera in the area of the removed pterygium with a fragment of the patient's conjunctiva, taken from another quadrant of the same or the other eye. This procedure is technically longer and more difficult than the simple excision method; however, it is also associated with a much lower postoperative recurrence rate. When harvesting a conjunctival graft, it is important to thoroughly remove Tenon's tissue because it is a source of fibroblast proliferation and a risk factor for pterygium regrowth. Reported rates of pterygium recurrence after conjunctival autografting range from 1% to approximately 40% [19]. For primary pterygium, many studies have reported recurrence rates of less than 15%, whereas for recurrent pterygium, recurrence rates range from 30 to 33% [14]. Absorbable sutures, tissue adhesives, and autologous patient blood can be used for conjunctival graft fixations. In the first case, the transplanted conjunctival flap is fixated with absorbable sutures, usually 8/0 Vicryl, which is associated with postoperative discomfort and irritation of the eyeball, while the most common complications of using tissue adhesive or autologous blood include graft dislocation. Recurrence rates may also vary depending on the fixation method used; however, there are conflicting reports in this regard. In a study by Sati et al. [12], there was no significant difference in pterygium recurrence among the three forms of conjunctival graft fixation. The study by Hall et al. [13], comparing the method using sutures with using tissue adhesive, showed a slightly higher recurrence rate when sutures were used (8.7% versus 0%, respectively). In a prospective randomized study comparing the tissue adhesive method with the method involving autologous blood [27], Nadarajah et al. obtained a slightly lower pterygium recurrence rate, at 3.4% in the 12-month follow-up for the tissue adhesive surgery group, and 10.6% for the autologous blood group. In our study, the autograft was fixed using absorbable sutures in all cases.

As the innermost layer of the placenta, the amniotic membrane is used for the treatment of pterygium owing to its unique structure. It consists of a single epithelial layer, together with a basal membrane and a stromal layer of extracellular matrix (ECM), consisting of a compact cell-free layer and a loose layer of fibroblasts [28]. Thanks to its biological properties, the amniotic membrane can be used as a graft with anti-inflammatory and antifibrotic properties, capable of delivering numerous growth factors and promoting epithelial cell proliferation and differentiation without the risk of immune reactions [29]. The use of amniotic membrane appears to be safe and effective and is associated with lower recurrence rates compared with the simple excision technique [30]. Reported recurrence rates with amniotic membrane transplantation range from 3.8% to 40.9%. In a prospective study, Prabhasawat et al. reported a recurrence rate of 10.9% after amniotic membrane transplantation [31]. Studies comparing the use of autograft with amniotic membrane transplantation (AMT) after pterygium excision have shown that AMT is associated with a higher recurrence risk at 6 months after surgery, compared with conjunctival autograft. AMT inferiority was observed in both primary and recurrent pterygium [32–38]. According to the authors, pterygium recurrence at 3 and 6 months after the surgery involving AMT ranges from 4.76% to 26.9% and from 2.6% to 42.3%, respectively. Amano et al. demonstrated a recurrence rate of 8.7% when mitomycin C was used intraoperatively (0.04%) [39]. Conjunctival autograft may be a source of conjunctival epithelium, while the amniotic membrane appears to play a role in inhibiting the progenitor cells involved in pterygium recurrence [40]. During the procedure, the amniotic membrane may be fixed with absorbable sutures or a tissue adhesive. This is the method of choice in cases of large pterygia, patients with postinflammatory conjunctival lesions, or in patients who may require future antiglaucoma surgery. When comparing the success rate of the procedure using conjunctival autograft versus the use of amniotic membrane, the data favored the conjunctival graft procedure at 7.4% versus 19.2% [41]. This was similar to the results obtained in our study (21.2% vs. 55.6%).

Our study has some limitations. First, it was a retrospective study, thus presenting lower evidence power in terms of its design. Second, the groups were not very large, and adjuvant treatment was not used. Furthermore, we have no information on the timing of exposure to factors that may promote postsurgical pterygium recurrence in patients. Nevertheless, this study was conducted on a homogeneous group of patients and is the first study on that subject in an Eastern European population.

## 6. Conclusion

Our study confirms that the conjunctival autograft surgeries present more favorable success rates in Polish Caucasian population when compared to procedures involving amniotic membranes. Further studies are needed to investigate the newer surgical techniques, finding their efficacy and long-term effects, as there are currently no reliable results in the literature. These techniques include limbal-conjunctival autografting and the combination of the limbal autografting method with simultaneous covering of the defect with amniotic membrane after pterygium excision.

## Figures and Tables

**Table 1:** Characteristics and comparison of groups (amniotic membrane vs. conjunctival autograft).

Variable	Amniotic membrane, *n* = 9	Conjunctival autograft, *n* = 52	MD/OR, 95% CI	*P*
Sex (female)	2 (22.2%)	19 (36.5%)	0.50 (0.09; 2.64)	0.479
Age	63.00 (51.00; 72.00)	62.00 (53.75; 70.25)	1.00 (−9.00; 9.00)	0.927
Observation period (months)	7.00 (3.00; 26.00)	19.00 (10.00; 29.25)	−12.00 (−15.00; 3.00)	0.127
Eye (right)	2 (22.2%)	17 (32.7%)	0.59 (0.11; 3.14)	0.707
Primary pterygium	3 (33.3%)	43 (82.7%)	0.10 (0.02; 0.50)	0.005
Recurring pterygium	6 (66.7%)	8 (15.4%)	11.00 (2.27; 53.27)	0.003
Other type	1 (11.1%)	1 (1.9%)	6.38 (0.36; 112.5)	0.275
Surgical success	4 (44.4%)	41 (78.8%)	0.21 (0.05; 0.94)	0.045

For quantitative variables, the median and quartiles 1 and 3 were reported; *n* (%) was reported for qualitative variables. MD/OR 95% CI, difference between medians (amniotic membrane minus conjunctival autograft) for quantitative variables or odds ratio for qualitative variables, with 95% confidence intervals. The odds ratio of an event is reported in the rows of the table.

## Data Availability

The data used to support the findings of this study are available from the corresponding author upon request.
